# CultureLED: A 3D printer-based LED illumination cultivation system for multi-well culture plates

**DOI:** 10.1016/j.ohx.2022.e00323

**Published:** 2022-05-31

**Authors:** Or Hasson, Asher Wishkerman

**Affiliations:** Faculty of Marine Sciences, Ruppin Academic Center, Mikhmoret 4029700, Israel

**Keywords:** 3D printing, Microalgae, Arduino, Cell culture, Cultivation, LED

## Abstract

Microalgae are a source of high value products such as pigments, lipids and carbohydrates. Microalgae cultivation techniques have evolved and improved, but a vast amount of research is still needed to achieve a better understanding of these microorganisms. Due to this, there is a growing need for affordable, flexible, and easy to control research systems and protocols. 3D printing revolutionized design and manufacturing as it became widely available to the mass market, allowing the creation of novel forms, enabling mass customization, and supporting low-volume, distributed production. The emergence of open-source designs combined with 3D printing applications have the potential to replace and outperform standard designs and methods. This protocol describes CultureLED, a well plated cell culture system that can be mounted on an orbital shaker or positioned on a shelf. It is an open hardware design, based on low-cost commercial off-the-shelf components. It was designed for optimized production cost, simplicity, low power consumption, design flexibility, and controllable light conditions. The CultureLED utilizes light-emitting diodes and it can be used for cultivation of small sized organisms or microorganisms with different light requirements and as such, has a wide range of applications.

## Graphical abstract

**Figure:**
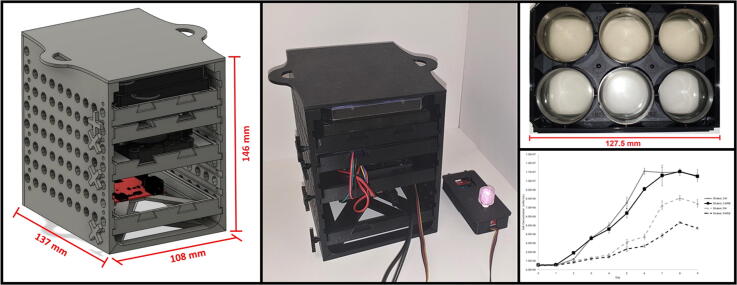

## Hardware in context

Research on microalgal biotechnology dates back to the 19th century and since then technologies have evolved and improved as innovative production systems such as photobioreactors (PBRs) have been employed to improve the algal biomass throughput per area [1]. In order to achieve a better understanding of these microorganisms and reach their maximum potential of applications and productivity, a vast amount of research of various factors is needed. Due to this, there is a growing need for affordable, flexible and easy to control research systems and protocols. This growing research effort is exemplified by the support of the European commission into research and development of microalgal biotechnology by allocating 40 million euros annually between 2007 and 2017 to microalgae-based ventures [2]. To date, there is a wide variety of cultivation systems and PBR types and designs [3]. Different PBR designs, volumes and shapes, allow different types of applications including cultivation systems and PBRs for microalgae mass commercial research and production, microalgae pure cultivation, and specialized PBRs designed for wastewater treatment, which are less prevalent [4]. Cultivation systems and PBRs for photoautotrophic prokaryotic and eukaryotic microorganisms use light (natural or artificial) as a primary energy source and exist in many forms (e.g., open vs closed, tubular vs panels, vertical vs horizontal) [5]. Light at a wavelength of 380–750 nm has the energy content to sufficiently produce chemical changes in the absorbing molecules (pigments). Some pigments are essential for photosynthesis including chlorophylls, carotenoids (carotenes and xanthophylls) and phycobilins. These pigments represent the three major classes of photosynthetic pigments in microalgae [6].

In recent years, the use of light-emitting diode (LED) based artificial light sources has increased due to their high electrical efficiency, low heat dissipation, good reliability, high durability, reasonable compactness, low cost and spectral output [[7], [8], [9]]. Among the different LED types, addressable Red, Green, Blue (RGB) LEDs have become popular as parameters like selected color, intensity and light/dark cycle are easy to control with minimal knowledge in programming and electronics. White light color is generated by a mix of three single primary colors RGB at maximum value. The relative intensity of the different color LEDs is easy to control via programming. The carbon source for photoautotrophic microorganisms is CO_2_ and it can be provided by shaking, stirring or via gas sparging to ensure microalgae cell mixing, and nutrient and gas exchange [10].

Microalgae are known to be a source of high-value products such as pigments, lipids, polysaccharides and other carbohydrates [11]. Cultivation of microalgae in photobioreactors is useful for producing these high value biochemicals [12]. These biochemicals can be used for a wide variety of applications such as food, cosmetics, medical, research and other uses, and it is believed that many important microalgal biochemicals are yet to be discovered [13]. The cell cultivation apparatuses vary in volumes and include for example: culture plates, Erlenmeyer flasks, bottles, tanks, or alternatively outdoor in tubular photobioreactors made from polyethylene sleeves or glass tubing. Nevertheless, in order to increase productivity, a basic understanding of photosynthesis, metabolism, and the mechanisms controlling cellular structure of microalgae using molecular genetics is indispensable. Although to approach these research objectives requires less medium volume and cultivation space as compared to production-scale systems. Indoor microalgae cultivation is usually done in climate-controlled growth rooms/cabinets where an orbital shaker is typically used for agitation. Small scale LED systems for microalgae cultivation mounted on orbital shakers are seldom published [[10], [14]] while publications regarding microalgae cultivation under different LED source light regimes are more common [[15], [16], [17], [18], [19]].

3D printing, a disruptive technology, revolutionized design and manufacturing as low-cost 3D printers became widely available to the mass-market allowing to create novel forms, enable mass customization and support low-volume and distributed production [20]. The commercialization and popularization of 3D printing yielded new and rapid prototyping and manufacturing approaches in a growing variety of fields and applications like in the case of a low cost, 3D-printed, open-source modular microscopy toolbox [21]. Another example is the incubot: a 3D printer-based, low-cost microscope, designed for inclusion within a conventional tissue culture incubator [22]. Similarly, the CultureLED design has an open-source nature, which allows adaptation and customization by both the community and individual researchers.

In this protocol we used 3D printed parts, 6-well transparent bottom cell culture plates, LEDs, DC Fans, SparkFun RedBoard with Qwiic connect system [23] (4-pin JST connectors) with additional components (temperature sensor, micro-OLED display, rotary encoder and motor driver). The advantage of the Qwiic system allows construction of daisy chain components without soldering, based on Qwiic Connect System (SparkFun Electronics, USA). This protocol describes an open hardware design, using low cost and commercial off-the-shelf components for an LED illuminated micro-plated cell culture system that can be mounted on an orbital shaker. It can be used for the cultivation of microorganisms or small sized organisms with different light requirements. The design parameters include an optimized production cost, simplicity, low power consumption, design flexibility and controllable light conditions. In summary, it is an economic build and an optimal platform for research and development, that could be further modified, updated and upscaled.

## Hardware description

**Fig. 1:**
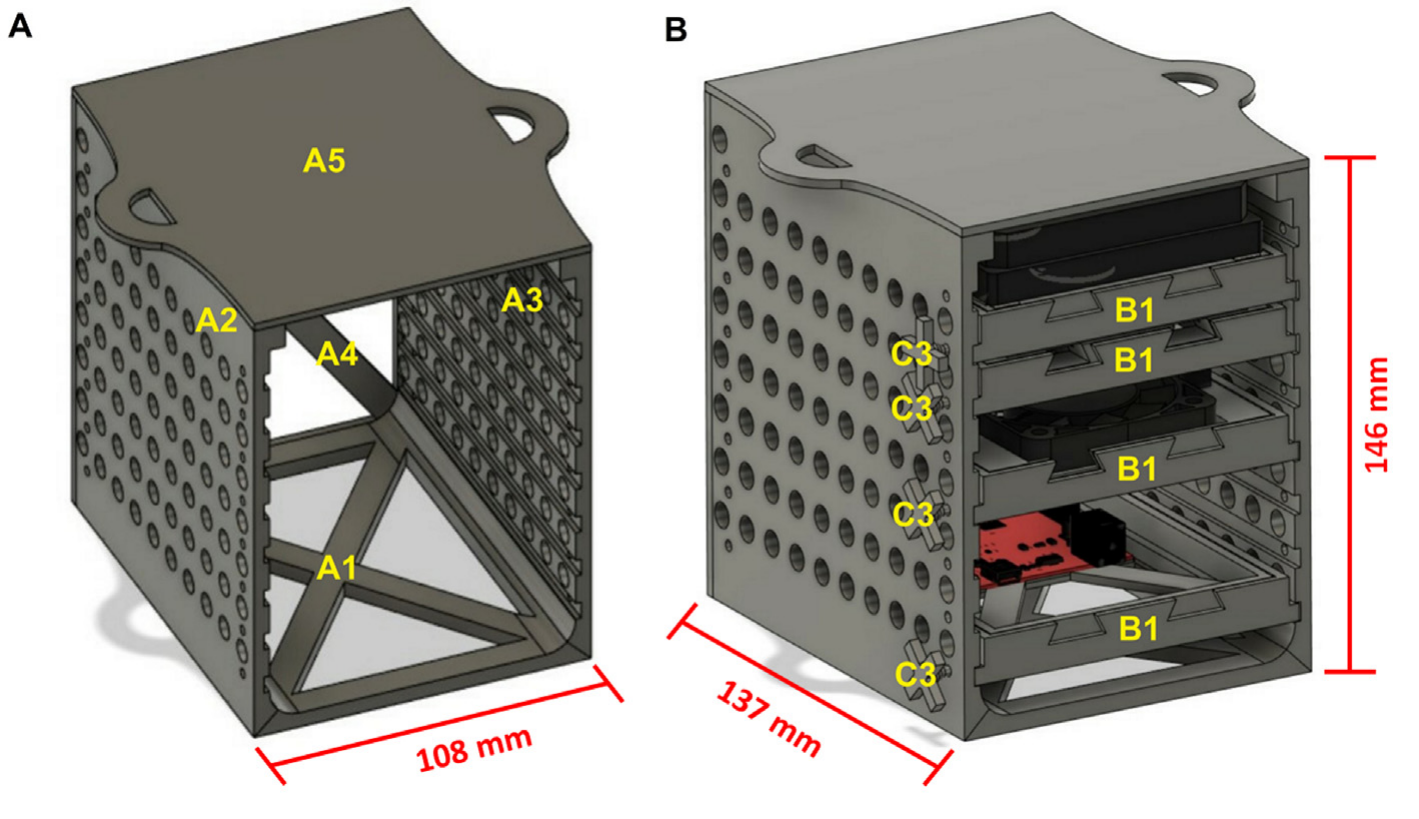
A) 3D rendered model of assembled CultureLED stand. B) 3D rendered of assembled stand with four drawers and locking pins. 3D parts labeled as followed: A1 - Base, A2 - Left Panel, A3 - Right Panel, A4 - Back Panel, A5 - Top Panel, B1 - Drawer, C3 - Locking Pins.

**Fig. 2:**
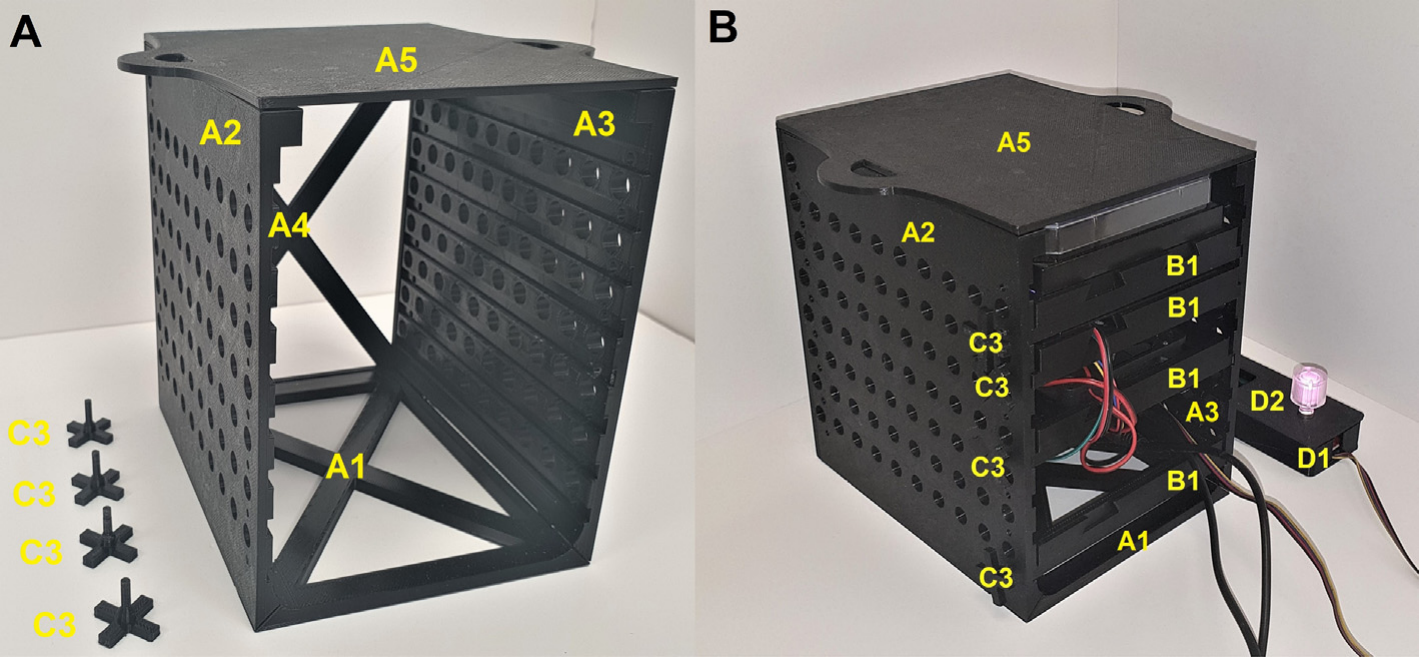
A) Photo of CultureLED assembled stand with locking pins. B) Photo of CultureLED assembled system with four drawers, inserted locking pins and a remote. Parts labeled as followed: A1 - Base, A2 - Left Panel, A3 - Right Panel, A4 - Back Panel, A5 - Top Panel, B1- Drawer, C3 - Locking Pins, D1 - Remote, D2 - Remote’s Lid.

**Fig. 3:**
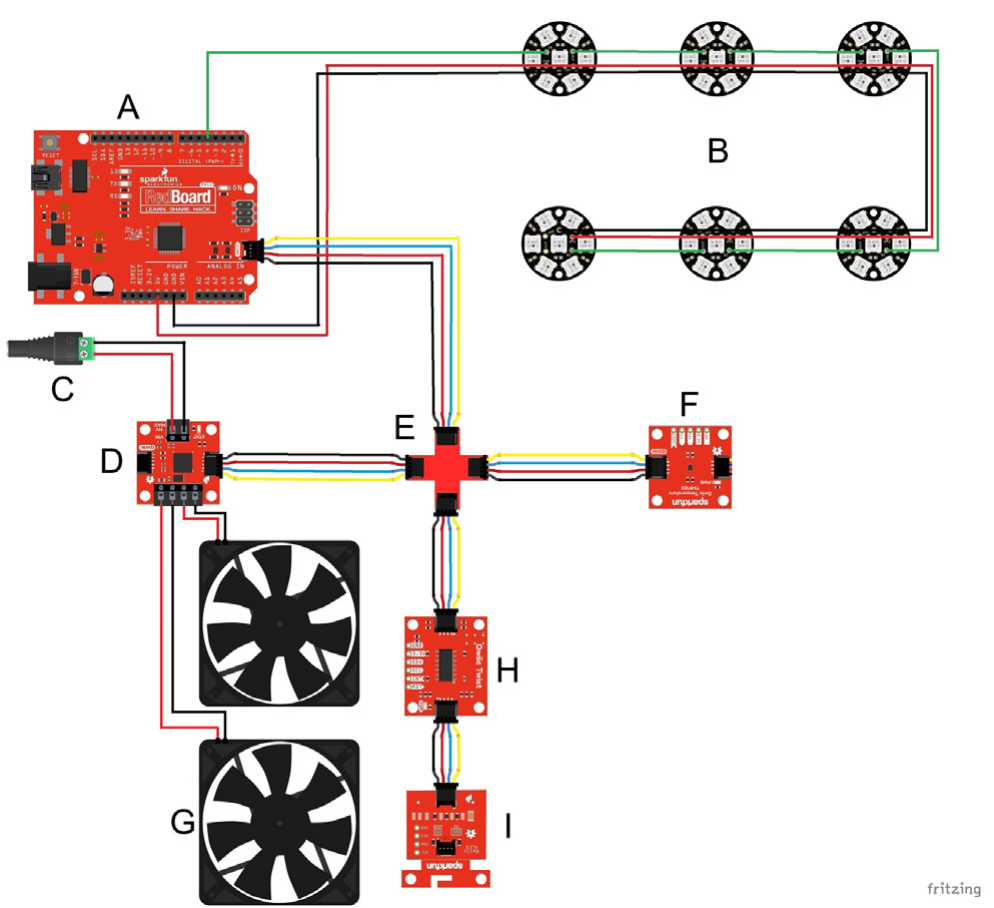
Wiring diagram. A) SparkFun RedBoard Qwiic; red, green and black jumper wires were used to connect the first LED to the board; B) Six units of NeoPixel Jewel; red, green and black hook-up wire were used to connect between the second and the sixth LEDs; C) Female DC Power adapter; D) Qwiic Motor Driver; E) SparkFun Qwiic MultiPort; F) Digital Temperature Sensor - TMP102 (Qwiic); G) Two units of 60 mm X 60 mm DC fans; H) Qwiic Twist - RGB Rotary Encoder Breakout; I) Micro OLED Breakout (Qwiic). Fritzing 0.8.7b was used to create this scheme (https://fritzing.org/releases/0-8-7b). Detailed Arduino script can be found in https://data.mendeley.com/datasets/jmg3nrvj9d/1. (For interpretation of the references to color in this figure legend, the reader is referred to the web version of this article.)

**Fig. 4:**
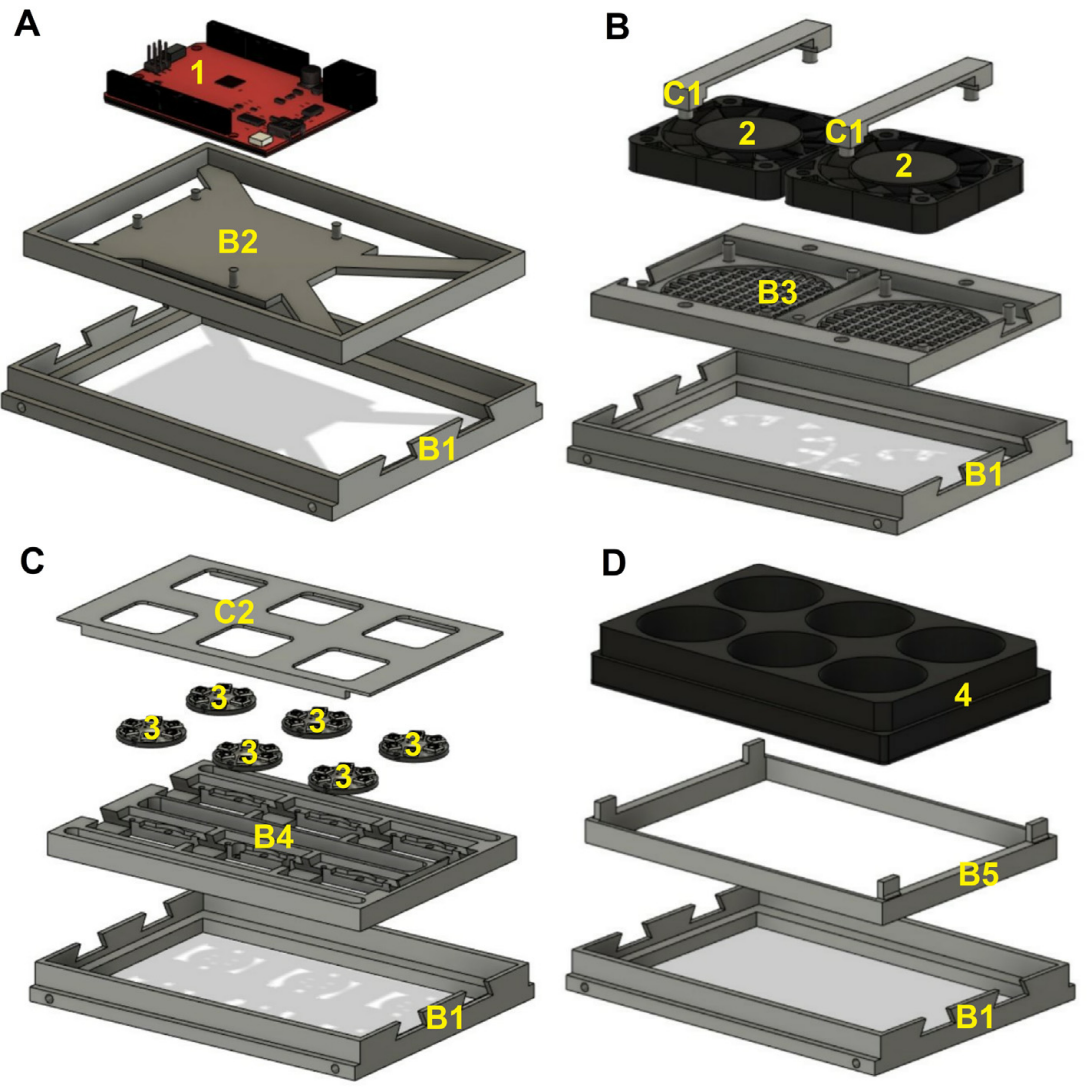
CultureLED four modular drawers and their components (3D rendered models): A) Drawer with the board panel, housing the RedBoard; B) Drawer with fans panel, fans and fan locks; C) Drawer with LED panel, LEDs and LED panel lid; D) Drawer with a 6 wells plate adapter and 6 wells plate on the top. Parts labeled as followed: B1 - Drawer, B2 - Board Panel, 1 - SparkFun RedBoard Qwiic, B3 - Fans Panel, 2 - DC fans, C1 - Fans Panel Locks, B4 - LED’s Panel, 3 - NeoPixel Jewel 7 units, *C*2 - LED’s Panel Lid, B5 − 6 Well Panel, 4–6 Well glass bottom plate.

**Fig. 5:**
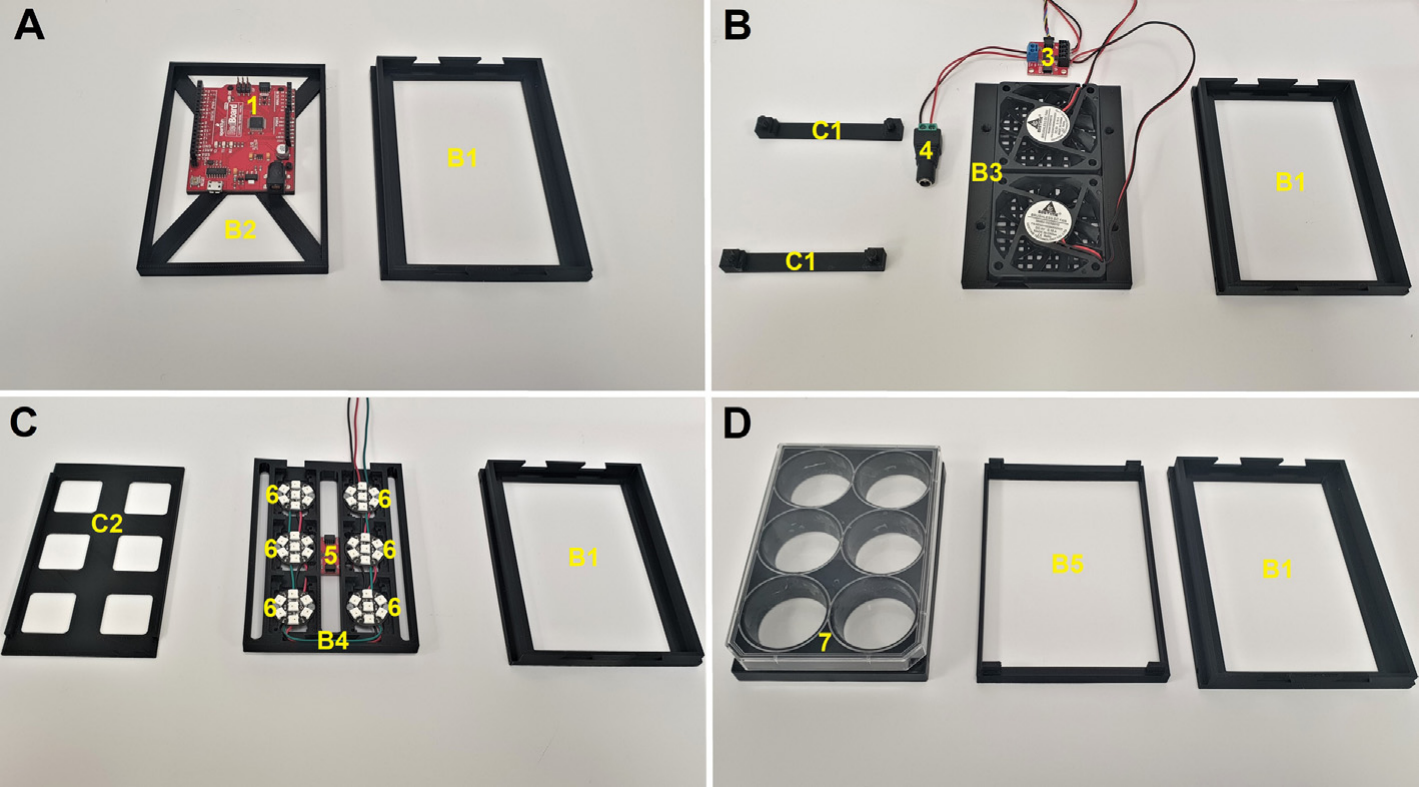
Photos of CultureLED four modular drawers and their components. A) Drawer with board panel, housing a RedBoard controller; B) Drawer with fans panel, fans and fan locks; C) Drawer with LED panel, LEDs and LED panel lid; D) Drawer with 6 wells plate adapter and 6 wells plate on the top. Parts labeled as followed: B1 - Drawer, B2 - Board Panel, 1 - SparkFun RedBoard Qwiic, B3 - Fans Panel, 2 - DC fans, C1 - Fans Panel Locks, 3 - Qwiic Motor Driver, 4 - Female DC Power adapter, B4 - LED’s Panel, 6 - NeoPixel Jewel 7 units, *C*2 - LED’s Panel Lid, B5 − 6 Well Panel, 7–6 Well glass bottom plate.

The modular drawer system is assembled from 3D printed parts (Polylactic acid, PLA) that are attached via printed plastic pins and can hold different components which include a 6-well transparent bottom cell culture plate, LEDs array panel, temperature sensor, two DC fans and SparkFun RedBoard Qwiic (Arduino-compatible development board) (Fig. 1, Fig. 2, Fig. 3, Fig. 4, Fig. 5). Addition and upgrade of components upon research requirements is possible as well as upscaling.

The LEDs array is based on six Adafruit NeoPixel Jewel units which are composed of seven 5050 WS2812B or SK6812 LEDs, for which each LED can be individually configurable (e.g., https://cdn-shop.adafruit.com/product-files/1138/SK6812+LED+datasheet+.pdf). Each NeoPixel is positioned below the 6-well transparent bottom cell culture plate that holds a microalgae culture. The illumination of the culture in terms of color, intensity and regime (e.g., continuous or flashing) can be controlled via programming and/or manually (described in section 6) while the light/dark cycle can be controlled by an external timer switch or programming. The default light intensity value of the system is 30 bit/54 µmol photons m^−2^ s^−1^ with a continuous white light program. Light intensities were measured using Apogee Quantum meter model MQ – 200 (Logan, USA). The light intensity is scaled by a bit range of 0–80 bits, above this range (up to 255) when very high light intensities are needed the system requires additional external power supply to be connected directly to the LEDs. Excess heat generated by the LEDs is removed by two 5 V DC fans controlled by the temperature sensor set value. The initial sensor value which operates the fans is set to 25° Celsius. This value could be modified in its section inside the Arduino INO code.

The CultureLED can be used for a wide range of applications and has several advantages: The light setup can be controlled via programming; it can be modified to support versatile light quality and quantity regimes. System dimensions are: Width: base 108 mm, top 148 mm; Depth: 137 mm; Height: 146 mm.

• The 6 well plate can be used to cultivate versatile photoautotrophic prokaryotic and eukaryotic microorganisms and small organisms with different medium or solid substrate requirements (e.g., Agar).

• The system is modular, easily assembled, and can be easily printed so it can fit into orbital shakers or shelves at different room temperatures.

• The small volume is ideal for performing molecular biology experiments.

• The compact nature of the design can potentially increase the number of simultaneous trials performed in parallel.

## Design files

The models were designed using Autodesk Fusion 360 software (Autodesk, USA).

**Design Files Summary**

**Table:** 

Design file name	File type	Open-source license	Location of the file
A1 – Base	STL	CC BY-SA 4.0	https://data.mendeley.com/datasets/jmg3nrvj9d/1
A2 – Left Panel	STL	CC BY-SA 4.0	https://data.mendeley.com/datasets/jmg3nrvj9d/1
A3 – Right panel	STL	CC BY-SA 4.0	https://data.mendeley.com/datasets/jmg3nrvj9d/1
A4 – Back Panel	STL	CC BY-SA 4.0	https://data.mendeley.com/datasets/jmg3nrvj9d/1
A5 – Top Panel	STL	CC BY-SA 4.0	https://data.mendeley.com/datasets/jmg3nrvj9d/1
B1 – Drawer	STL	CC BY-SA 4.0	https://data.mendeley.com/datasets/jmg3nrvj9d/1
B2 – Board Panel	STL	CC BY-SA 4.0	https://data.mendeley.com/datasets/jmg3nrvj9d/1
B3 – Fans Panel	STL	CC BY-SA 4.0	https://data.mendeley.com/datasets/jmg3nrvj9d/1
B4 – LED’s Panel	STL	CC BY-SA 4.0	https://data.mendeley.com/datasets/jmg3nrvj9d/1
B5 – 6 Well Panel	STL	CC BY-SA 4.0	https://data.mendeley.com/datasets/jmg3nrvj9d/1
C1 – Fans Panel Locks	STL	CC BY-SA 4.0	https://data.mendeley.com/datasets/jmg3nrvj9d/1
*C*2 – LED’s Panel Lid	STL	CC BY-SA 4.0	https://data.mendeley.com/datasets/jmg3nrvj9d/1
C3 – Locking Pins	STL	CC BY-SA 4.0	https://data.mendeley.com/datasets/jmg3nrvj9d/1
D1 + D2 – Remote + Remote’s lid	STL	CC BY-SA 4.0	https://data.mendeley.com/datasets/jmg3nrvj9d/1
CultureLED Operation - Arduino Sketch	INO	CC BY-SA 4.0	https://data.mendeley.com/datasets/jmg3nrvj9d/1

### A1 – Base

The base part holds the entire system. The left, right and back panels (parts A2, A3, and A4, respectively) will be connected to this base with their printed pins.

### A2 and A3 – Left and right panels

The panels are connected to the base, back panel, and top panel via their printed pins. These parts include paths for the component’s drawers as well as small holes (3.2 mm diameter) to lock the drawers. An array of holes (7.5 mm diameter) allows ventilation and heat exchange as well as safer passing of cables if needed.

### A4 – Back panel

The back panel is connected to the base and the side panels. Its aim is to provide support and strength to the structure and stop the drawers from sliding out.

### A5 – Top panel

The top panel is sealing the structure from above preventing light from entering and going out from the system. It also strengthens the system's structure. The top panel is connected to the left and right panels throughout its 6 printed pins, 3 to each side panel, and designed with 2 side handles for easy disassembly.

### B1 – Drawer

The drawer is a modular part that can hold all the components panels (B parts). The drawer slides in through the grooves of the left and right panel (parts A2 and A3) and can be locked using the locking pins (part C3). The drawer also features four trapezoid entrances for cable access and rearrangements.

### B2 – Board panel

The board panel is designed to house the SparkFun RedBoard QWIIC board of the system. It has 4 pins to mount the board. This panel fits inside the drawer (part B1) and it’s the first panel from below.

### B3 – Fans panel

The fans panel is designed to house two 60 mm X 60 mm DC fans, to dissipate possible heat released during the LED’s activity. The panel has small trapezoid paths for cables and 4 top holes that connect to 2 bridge lockers. This panel is sitting on a drawer, and it’s the second panel from below.

### B4 – LED’s panel

The LED’s panel is designed to hold six Adafruit NeoPixel Jewel LED units, one below each well of the 6 well plate. The LED units are connected to the panel via screws. Furthermore, the temperature sensor is mounted below via screws. This panel is sitting inside a drawer and located below the 6 well plate. It’s the third panel from below.

### B5 – 6 well panel

This panel is simply designed to hold a 6 well plate, without blocking the emitted light below it. It is compatible with several 6 well plate brands although we used Cellvis 6 Well glass bottom plate with high performance #1.5 cover glass due to its black frame. This panel is sitting on a drawer, and it should be located above the LEDs panel (part B4) and below the top panel (part A5).

### C1 – Fans panel locks

These locks are designed to hold the fans to their panel via pins with minimal disturbance to the airflow. Each lock has a small step to route the fan cables.

### C2 – LED’s panel lid

The lid is designed to cover the LED’s panel and protect them and their wiring while providing a better aesthetic look.

### C3 – Locking pins

The locking pins are small parts that lock and the drawers to the left and right panels (parts A2 and A3). These pins are designed to be inserted into the small holes in the side panels into the drawers.

### D1 + D2 – Remote + Remote’s lid

The remote is an additional and external part that is designed to hold two electronic parts: Micro OLED Breakout (Qwiic) and the Qwiic Twist - RGB Rotary Encoder Breakout. This part is connected to the RedBoard via a long Qwiic cable (500 mm) and allows the user to change predefined light programs, control the lighting intensity and display the temperature around the LEDs.

## Bill of materials

**Table:** 

Designator	Component	Number	Cost per unit - USD	Total cost - USD	Source of materials	Material type
DEV-15123 or DEV-18158	SparkFun RedBoard Qwiic or SparkFun RedBoard Plus	1	$19.95	$19.95	https://www.sparkfun.com/products/15123	*Other*
DEV-15083	Qwiic Twist - RGB Rotary Encoder Breakout	1	$22.95	$22.95	https://www.sparkfun.com/products/15083	*Other*
SEN-16304	Digital Temperature Sensor - TMP102 (Qwiic)	1	$6.50	$6.50	https://www.sparkfun.com/products/16304	*Other*
LCD-14532	Micro OLED Breakout (Qwiic)	1	$16.95	$16.95	https://www.sparkfun.com/products/14532	*Other*
COM-10597	Clear Plastic Knob	1	$0.95	$0.95	https://www.sparkfun.com/products/10597	*Other*
ROB-15451	Qwiic Motor Driver	1	$17.95	$17.95	https://www.sparkfun.com/products/15451	*Other*
2226	NeoPixel Jewel − 7 × 5050 RGB LED with Integrated Drivers	6	$5.95	$35.70	https://www.adafruit.com/product/2226	*Other*
PRT-14426	Qwiic Cable – 50 mm	1	$0.95	$0.95	https://www.sparkfun.com/products/14426	*Other*
PRT-14428	Qwiic Cable – 200 mm	3	$1.50	$4.50	https://www.sparkfun.com/products/14428	*Other*
PRT-17257	Qwiic Cable – 500 mm	1	$1.95	$1.95	https://www.sparkfun.com/products/17257	*Other*
BOB-18012	SparkFun Qwiic MultiPort	1	$1.95	$1.95	https://www.sparkfun.com/products/18012	*Other*
PLA-0.5	Flashforge PLA 1.75 mm 3D Printer Filaments 0.5 kg Spool	1	$17.99	$17.99	https://www.flashforgeshop.com/product/pla-filament-500g-black	*Polymer*
1351	2-Way 2.1 mm DC Barrel Jack Splitter Squid	1	$2.95	$2.95	https://www.adafruit.com/product/1351	*Other*
368	Female DC Power adapter − 2.1 mm jack to screw terminal block	1	$2.00	$2.00	https://www.adafruit.com/product/368	*Other*
	Generic 9 V 3A DC Power Supply	1	$4.23	$4.23	https://www.aliexpress.com/item/4000433119623.html	*Other*
GDA6010 Axial Fan	DC fan	2	$1.50	$3.00	https://www.alibaba.com/product-detail/Gdstime-GDA6010-DC-5V-12V-24V_62527011003.html	*Other*
PRT-11026	Jumper Wires Standard 7″ M/M − 30 AWG (30 Pack)*	1	$2.25	$2.25	https://www.sparkfun.com/products/11026	*Other*
PRT-11375	Hook-Up Wire - Assortment (Stranded, 22 AWG)**	1	$20.95	$20.95	https://www.sparkfun.com/products/11375	*Other*
P06-1.5H-N	6 Well glass bottom plate with high performance #1.5 cover glass***	1	$11.55	$11.55	https://www.cellvis.com/_6-well-glass-bottom-plate-with-high-performance-number-1.5-cover-glass_/product_detail.php?product_id=55	*Other*
	M2x6 mm plastic screws	12	$0.07	$3.69	https://www.aliexpress.com/item/1005002614020555.html	*Polymer*
	Grand Total			$198.91		

* Not all case content is needed.

** Some extra wires are needed for soldering, not all case content is needed.

*** Sold in case of 20 units, only one is use for each CultureLED. Cost calculation is based on one plate.

## Build instructions

### Preassembly safety note:

The assembly of this system requires 3D printed parts and their mechanical assembly (sections 5.1–5.4), minimal soldering and connection of the electronic parts (section 5.5) and programming (section 5.6). Take any necessary precautions while working with the 3D printer and solder to prevent short circuits or injury. Take care while working with electricity.

### Printing configuration:

The PLA based parts were printed using Flashforge Adventurer 3 printer (Zhejiang Flashforge 3D Technology Co., ltd., China) using Flashprint software (version 4.6.2), with the following settings: nozzle size: 0.4 mm, extruder temperature: 210 °C, platform temperature: 50 °C, layer height: 0.18 mm, first layer height: 0.27 mm, perimeter shells: 2, top solid layers: 4, bottom solid layers: 3, fill density: 15 %, fill pattern: hexagon, combine infill: every 2 layers, print speed: 60 mm/s, travel speed: 80 mm/s, cooling fan always on. No wall, raft or brim was added to the models.

With exception to the above, Part C3 – Locking Pins were printed with the same parameters above but with fill density: 100 % due to their small size.

### Stand assembly (parts A1 to A5):

The 3D printed parts build instructions are also shown in an animation file (CultureLED_assemblyGuide.MP4) and movie (CultureLED_systemOperation.mp4). The animation file was done using Autodesk Fusion 360 software (Autodesk. USA) and Corel VideoStudio Ultimate 2021 (Corel Corporation, Canada).

Step 1:

Place the base panel (Fig. 1A, part A1) on a solid, balanced surface, then take both side panels (Fig. 1A, parts A2 and A3) and connect them using their pins. Make sure that the panels are placed in the right direction, the base panel two horizontal rounded holes should be located in the back of the structure. Apply a little pressure to hold them tighter to the base.

Step 2:

Once parts A1, A2, and A3 are assembled in the right direction, connect the back panel (Fig. 1A, part A4) with its pins to the four rounded holes in the back of the assembly (step 1). Please be aware that the smaller holes should be located at the top.

Step 3:

Take the top panel (Fig. 1A, part A5) and connect it to the top of the structure using its six printed pins, three connected to each side. Apply a little pressure from all sides to tighten all the parts together and avoid unnecessary spaces as shown in Fig. 1A and Fig. 2A.

### Drawers and components assembly (parts B1 to B5):

Once section 5.1 is completed, the assembly/stand is ready for the modular drawer including the components that fit in it. Before continuing, make sure to properly connect and solder the electronic parts as shown in Fig. 3. Also, notice that this section requires four printed units of the drawer part (Fig. 1B and 2B, parts B1) and locking pins (Fig. 1B and 2B, parts C3).

Step 1:

Connect the SparkFun RedBoard Qwiic (Fig. 4A, part 1) to a board panel (Fig. 4A, part B2) using the four mounting holes. The board DC jack and USB connector should be facing forward (Fig. 4A and Fig. 5A). Insert this assembly into one drawer (Fig. 4A, part B1) and put it aside until the final assembly.

Step 2:

Attach two 60 mm × 60 mm × 10 mm DC fans where their cables face forward to the fans panel (Fig. 4B and 5B, part B3). Mount the fans panel locks (Fig. 4B and 5B, part C1) to the fans panel (Fig. 4B and 5B, part B3) to lock them. Make sure it’s well placed over each fan, while the cables run smoothly underneath them, in the dedicated path. Screw the fan power cables to the Qwiic Motor Driver (Fig. 5B, part 3). Insert this assembly into another drawer (Fig. 4B and 5B, part B1) and slide it to the stand as shown in Fig. 4B.

Step 3:

Screw to the bottom of the LED panel (Fig. 4C and 5C, part B4) a digital temperature sensor - TMP102 (Qwiic) (Fig. 5C, part 5) using M2x6mm plastic, the sensor should be faced up (as shown in Fig. 5C, part 5). Make sure that the TMP 102’s Qwiic cable is connected from below. Now, place the soldered Adafruit NeoPixel Jewel LEDs and the attached wires (section 5.5), screw the LEDs using with two M2x6mm for each unit, except LEDs located at position #2 and #4 (in the row) that are close to the sensor, each should be connected with only one screw (Fig. 5C, parts B4 and 6). Put the LED’s panel lid (Fig. 5C, part C2) on the LED’s panel (Fig. 5C, part B4) and make sure it is not obscuring the LEDs. Insert this assembly into another drawer (Fig. 5C, part B1) and slide it into the stand as shown in Fig. 4C. The wires should face outside the drawer and later will be connected to the RedBoard (section 5.5).

Step 4:

Attach a 6 well plate (Fig. 4D, part 4 and Fig. 5D, part 7) with microalgae culture to the 6 well panel (Fig. 4D and 5D, part B5) and gently tighten and align it where the 4 pins are supporting it. Insert this assembly into another drawer (Fig. 4D and 5D, part B1) and slide it into the stand as shown in Fig. 4D and Fig. 5D. In case that sterile conditions are needed, considering the nature of the microorganisms to be cultured, assemble this part in a laminar flow or a biological safety cabinet.

After the first assembly, it is recommended to install this part as the last step before the experimental phase starts. Be aware that the advised working volume should be 7.5–10 ml if the CultureLED will be installed on an orbital shaker.

### Locking and final positioning (part C3):

Step 1:

Connect a Qwiic MultiPort to a 200 mm Qwiic cable which is connected to a RedBoard. Now, connect the Qwiic cables from the temperature sensor and motor driver to the MultiPort. The LED wires (data, 5 V, and ground) should be connected to their proper pins (section 5.5). Slide all four drawers with their components in the following order from bottom to top, as shown in Fig. 1B and Fig. 2B:

1. Board panel (parts B1 and B2).

2. Fans panel (parts B1 and B3).

3. LED’s panel (parts B1 and B4).

4. 6 Well panel (parts B1 and B5).

Step 2:

After sliding the drawers, lock each drawer to its place with the locking pins (Fig. 2B, part C3) by inserting them into their dedicated holes in the side panels (Fig. 2B, parts A1 and A2).

### Remote assembly (parts D1 to D2):

**Fig. 6:**
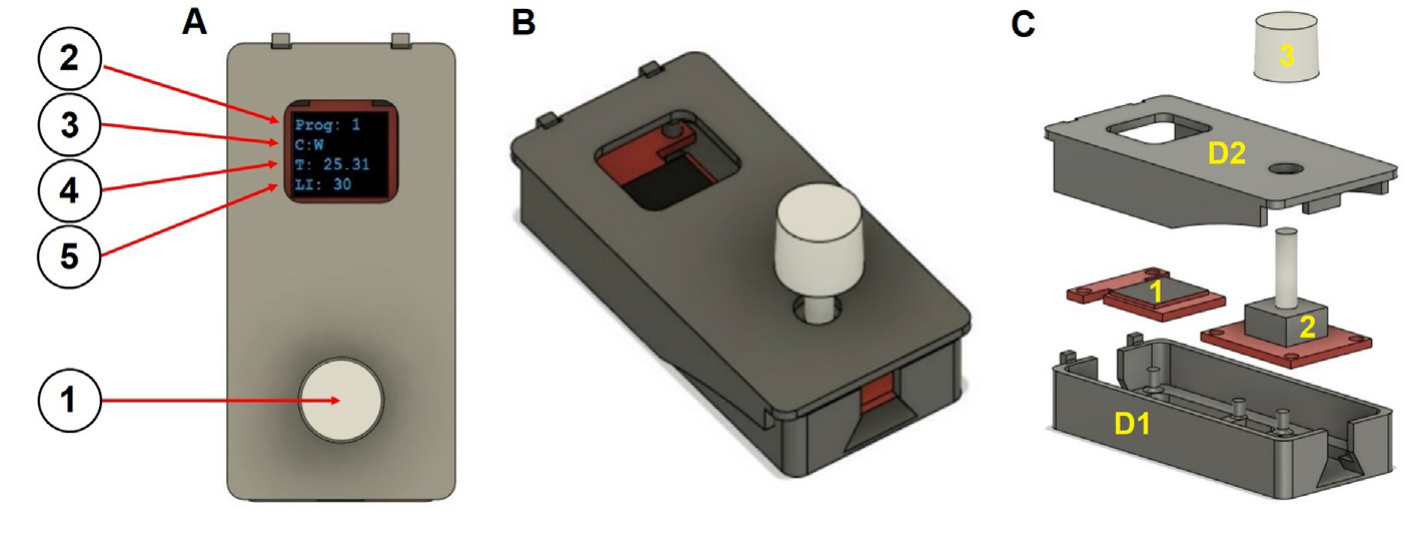
3D rendered models. A) System remote in top view. 1) Remote button. 2) Program number indication. 3) Program name indication. 4) Temperature indication. 5) Light intensity indication; B) System remote in isometric view; C) Components that assemble the remote. Parts labeled as followed: D1 - Remote, D2 - Remote’s Lid, 1 - OLED Breakout (Qwiic), 2 - Qwiic Twist, 3 - Twist clear plastic knob.

The system remote designs are shown in Fig. 6A, B and C.

Step 1:

Connect a Micro OLED Breakout (Qwiic) (Fig. 6C, part 1) to a Qwiic Twist - RGB Rotary Encoder Breakout (Fig. 6C, part 2) with a 50 mm Qwiic cable. Use a 500 mm Qwiic cable to connect the Qwiic Twist - RGB Rotary Encoder Breakout to the SparkFun Qwiic MultiPort, as shown in the scheme (Fig. 3).

Step 2:

Place the step 1 assembly to the remote (Fig. 6C, part D1) on top of the pins as shown in Fig. 6C and apply minimal pressure.

Step 3:

Put the lid (Fig. 6C, part D2) on while the rounded hole is above the Twist - RGB Rotary Encoder Breakout as shown in Fig. 6C.

Step 4:

Once the lid is closed, attach the SparkFun Clear Plastic Knob (Fig. 6C, part 3) to the Qwiic Twist - RGB Rotary Encoder Breakout (Fig. 6C, part 2) to lock the lid. The remote assembly is shown in Fig. 6.

### Electronic Scheme:

The wiring diagram (Fig. 3) presents the electronic layout of the components. The parts should be connected and soldered accordingly.

### Arduino INO sketch:

The code should be uploaded to the SparkFun RedBoard using the USB connector (more information can be found in https://learn.sparkfun.com/tutorials/redboard-qwiic-hookup-guide/all).

## Operation instructions

**Fig. 7:**
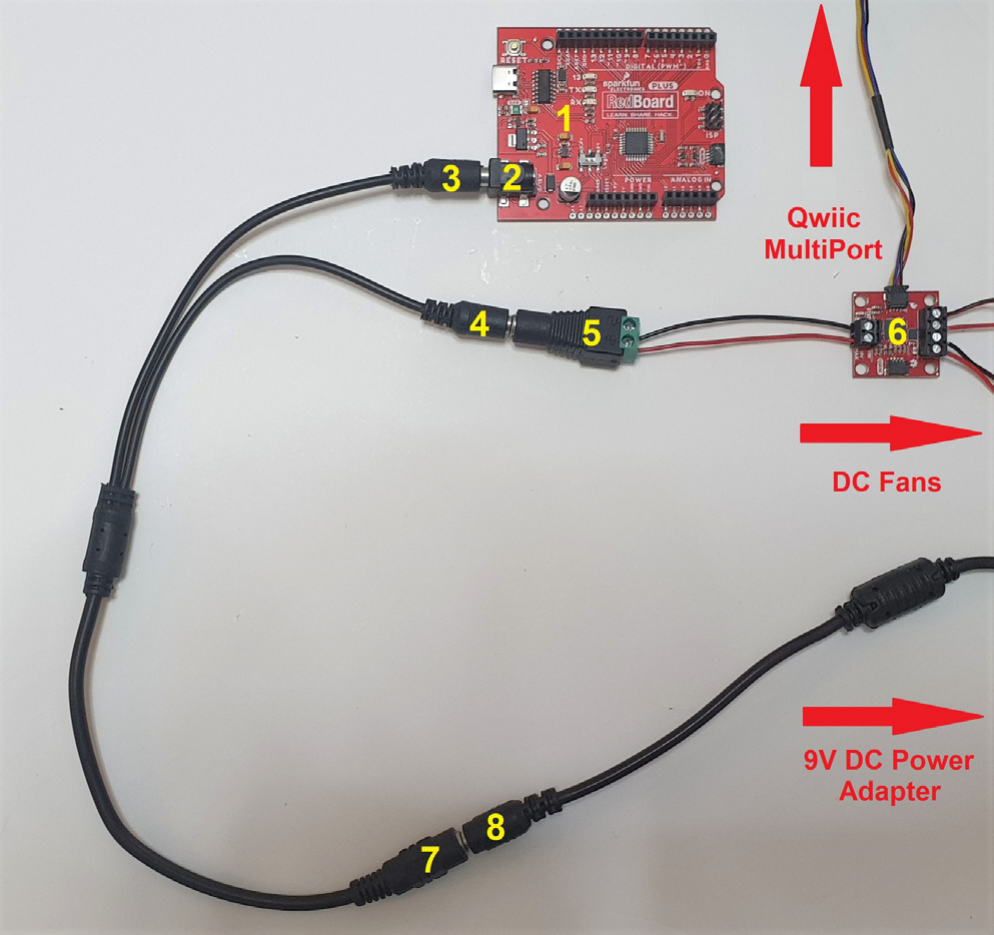
Photo of the 2-Way 2.1 mm DC Barrel Jack Splitter Squid assembly, which connects the system to the 9 V 3A DC Power Supply. Parts labeled as followed: 1 - SparkFun RedBoard, 2 – RedBoard DC 2.1 mm jack (female), 3 - First DC Barrel Splitter Squid’s jack (male), 4 - Second DC Barrel Splitter Squid’s jack (male), 5 - Female DC Power adapter − 2.1 mm jack to screw terminal block, 6 - SparkFun Motor Driver, 7 - Third DC Barrel Splitter Squid’s jack, DC power adapter end (female). 8 - DC power adapter 2.1 mm jack (male).

Once the system’s assembly described in section 5 is complete, use a 2-Way 2.1 mm DC Barrel Jack Splitter Squid to provide DC power to both the RedBoard and the Qwiic Motor Driver. Connect to the RedBoard (Fig. 7, part 1) DC jack (female) (Fig. 7, part 2) one end of the splitter (male) (Fig. 7, part 3). Connect the second end (male) (Fig. 7, part 4) to a Female DC Power adapter − 2.1 mm jack (Fig. 7, part 5). The Female DC Power adapter’s screw terminal block should be connected to the Qwiic Motor Driver (Fig. 7, part 6) screw terminal block. The 2 DC fans and the Qwiic multiport should be connect to the Qwiic Motor Driver (Fig. 7, part 6) as indicated by the arrows. Connect the main splitter squid (female) (Fig. 7, part 7) to a 9 V DC power supply (male) (Fig. 7, part 8).

### Remote operation

The remote allows an easy selection of stored light regime programs and intensities as well as monitoring the current temperatures. The remote is simply designed with one button (Qwiic Twist - RGB Rotary Encoder) that can be pressed in order to switch light program or twisted clockwise and counterclockwise in order to adjust the light intensity. The remote scheme is shown in Fig. 6A and described below.

Remote scheme description:

**Remote button** - The button can be pressed to change a stored program or rotated clockwise/counterclockwise to change the light intensity. (Fig. 6A.1).

**Light program number** - The stored and selected programs can be changed by pressing the remote button (A), as explained in the INO file (Fig. 6A.2).

**Light program name** - Each program has a number and a brief description (Fig. 6A.3, as programmed directly in the INO file). For example, in our code (CultureLED_CodeFeb2022.ino):

Program #1: “C:W”- C indicates continuous mode while W stands for white led color. This is the default mode.

Program #2: “C:W/WRB”- C indicates continuous mode while W/WRB, stands for LED’s coloration. In this case, W indicates white, WRB indicates a mix of white, red and blue, R indicates red and B indicates blue. Our setup was 6 LEDs:3 LED W and 3 WRB.

Program #3: “F:W/WRB”- F indicates flashing mode. The flashing time is indicated by the delay function set to 1000 ms. The LED color setup is like Program #2.

**Temperature -** The temperature reading output is shown in Celsius (default), but it can be changed to Fahrenheit as explained in the INO file (Fig. 6A.4).

**Light intensity -** The display shows the light intensity value (0–80 bits) (Fig. 6A.5).

### Routine system operation:

Once the system is assembled, every part can be unlocked by simply removing its locking pin (Fig. 2A, part C3) and sliding it out from the stand. Nevertheless, in routine, the 6 well panel will be replaced more frequently. Other parts are likely to be taken out only during maintenance, between experiments, or in disassembly.

## Validation and characterization

### Microalgae cultivation:

**Fig. 8:**
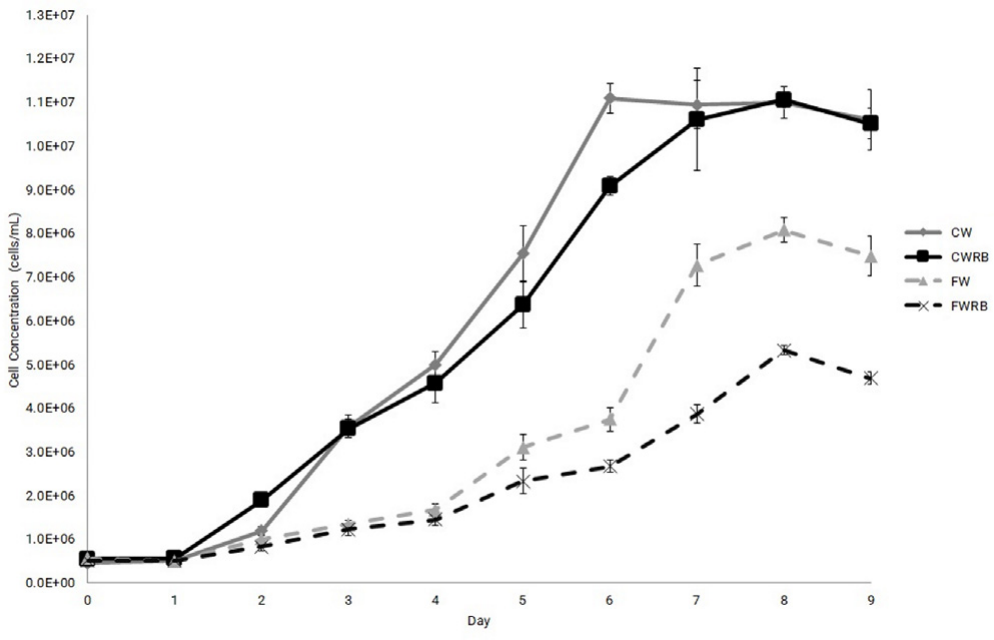
Growth of *P. tricornutum*, cultured under continuous white light (CW), continuous white, red and blue lights (CWRB), flashing white light (FW) and flashing white, red and blue lights (FWRB). Error bars represent standard deviation, n = 3. (For interpretation of the references to color in this figure legend, the reader is referred to the web version of this article.)

The validation of the system was done by two CultureLED systems that each contain one 6 well plate. The cultivation of *Phaeodactylum tricornutum* has been performed with an initial culture volume of 7.5 ml per well of artificial seawater - F/2 medium [24]. The CultureLED was installed on an orbital shaker (Digital Orbital Shaker, Heathrow Scientific, USA) set to a rotation speed of 75 RPM, installed inside a cooling incubator set to 25 °C and under ambient CO_2_ conc. (414 ppm). A set of three wells were treated with different light regimes, e.g., continuous white (CW); continuous white, red and blue (CWRB); flashing white (FW), and flashing white, red and blue (FWRB) (Fig. 8). The light intensities were 54 µmol photons m^−2^ s^−1^ in CW and FW regimes, and 33 µmol photons m^−2^ s^−1^ in CWRB and FWRB regimes. The light/dark period in flashing light regimes (FW, FWRB) was 1 s on/1 s off (0.5 Hz). Daily growth and growth rates were achieved by counting cells from each well, using a hemocytometer that contains quadrilateral gridded chambers with known volume, which enables cell quantification in a specific volume. The duration of the cultivation experiment was 9 days.

### Cell growth rate results

As shown in Fig. 8, significant cell growth has been achieved for the cultures cultivated under continuous LED light, compared to flashing LED light. White light-based regimes (CW and FW) produced better growth than white, red, and blue light-based regimes (CWRB and FWRB). *P. tricornutum* growth was better in continuous regimes compared to flashing regimes. The average maximum cell concentration in the CW regime was achieved at day 6 (11.1 × 10^6^ cells/ml) while the maximum average cell concentrations in CWRB, FW, and FWRB regimes were achieved two days later (day 8) and were 11.0 × 10^6^, 8.1 × 10^6^ and 5.3 × 10^6^ cells/ml, respectively. Throughout the experiment period, it was observed that *P. tricornutum* grew better under continuous light regimes (e.g., up to ∼ 2.3 times more cells/ml on the last day of the experiment) (Fig. 8). Similar results have been achieved with *Chlorella vulgaris*, which was grown under 1 s/ 1 s (0.5 Hz) light/ dark LED light regimes, compared to continuous LED light regimes [18]. Nevertheless, various algal strains show different growth rates in other light regimes as shown in several publications [[15], [16], [17], [19]]. We hope that the system users will examine other light regimes and different algal strains.

## Conclusions

The protocol presents a 3D printer-based LED illumination cultivation system for multi-well culture plates that can be mounted on an orbital shaker or shelf. The bill of materials, assembly instruction and code allow third-party customization for different purposes or experimental requirements. The system parts can be printed and assembled within days using low-cost commercially available off-the-shelf materials, allows updating of electronic parts during their life cycle and implanting improvements and modifications. The modular drawer design is flexible allowing exchange of the panels, by this it is possible to use different development boards, other multi-well plates (e.g., smaller volume wells), various LEDs and fans. The system can be located inside a growth chamber or growth room to allow temperature, humidity, and/or CO_2_ control based on the experimental parameters.

## Human and animal rights

Not applicable.

## Declaration of Competing Interest

The authors declare that they have no known competing financial interests or personal relationships that could have appeared to influence the work reported in this paper.
